# Multisource feedback in residency training: A quantitative study to investigate the feedback conversation

**DOI:** 10.3205/zma001791

**Published:** 2025-11-17

**Authors:** Eva K. Hennel, Felicitas-M. Lahner, Noemi Zweifel, Sigrid Harendza, Kathrin Neuhaus, Sören Huwendiek

**Affiliations:** 1 University of Bern, Institute for Medical Education, Department for Assessment and Evaluation (AAE), Bern, Switzerland; 2 Schweizerisches Institut für ärztliche Weiter- und Fortbildung SIWF, Bern, Switzerland; 3 University of Applied Sciences, Department of Health Professions, Bern, Switzerland; 4 University Children’s Hospital Zürich, Department of Surgery, Zürich, Switzerland; 5 University Medical Centre Hamburg-Eppendorf, III. Department of Internal Medicine, Hamburg, Germany

**Keywords:** professional identity formation, formative assessment, multisource feedback, residency training, workplace-based assessment

## Abstract

**Introduction:**

Multisource Feedback (MSF) is one form of assessment in medical training. It provides individual feedback based on multiple ratings, which can then be used to develop learning goals during a feedback conversation between supervisor and resident. We conducted this study to investigate how the resident and supervisor set the learning goals and what the learning goals refer to.

**Methods:**

The study comprised 75 sets of MSF, each consisting of 12-15 external ratings per resident and one self-rating per resident. Data included 1015 external ratings and 75 self-ratings. As ten sets missed learning goals, only 65 sets of MSF could be analysed. Data comprised written MSF feedback, including scale-based ratings and narrative comments, structured minutes from the feedback conversations and the resulting learning goals, which were sorted into themes. We used multiple linear regressions to determine the associations between feedback data, conversation topics and learning goals.

**Results:**

Topics were more likely to be discussed as strengths during the feedback conversation if scale-based ratings were high and there were many favourable comments on an item. Topics were more likely to be discussed as areas for improvement if the number of unfavourable comments was high. Topics with many (favourable and unfavourable) comments and topics discussed as an area for improvement were more likely to result in learning goals. We found a number of learning goals beyond the competences on the MSF-questionnaire, that can be understood as connected to Professional Identity Formation.

**Conclusion:**

As the feedback and learning goals clearly exceeded the expected competences from the MSF questionnaire, we see the need for addressing these broader topics of residents’ development. Hence, we encourage supervisors and residents to explicitly include Professional Identity Formation topics such as career plans, engagement in research or personal attitudes into regular assessments and feedback conversations. Thus, MSF might be a fitting tool to support professional identity development.

## Zusammenfassung

**Einleitung:**

Multisource Feedback (MSF) ist ein Bewertungsformat in der medizinischen Aus- und Weiterbildung. Es liefert individuelles Feedback auf Grundlage mehrerer Bewertungen, das dann in einem Feedbackgespräch zwischen der Supervisionsperson und der Person in Weiterbildung zur Entwicklung von Lernzielen verwendet werden kann. Wir haben diese Studie durchgeführt, um zu untersuchen, wie Weiterzubildende und Supervisionspersonen die Lernziele festlegen und worauf sich diese Lernziele beziehen.

**Methoden:**

Die Studie umfasste 75 MSF-Datensätze, die pro Person in Weiterbildung aus 12 bis 15 externen Bewertungen und einer Selbstbewertung bestanden. Die Daten umfassten insgesamt 1015 externe Bewertungen und 75 Selbstbewertungen. Da zehn Datensätze keine Lernziele enthielten, konnten nur 65 MSF-Datensätze analysiert werden. Die Datensätze enthielten schriftliches MSF-Feedback, einschliesslich skalenbasierter Bewertungen und narrativer Kommentare sowie strukturierte Protokolle der Feedbackgespräche mit den daraus resultierenden Lernzielen, die nach Themen sortiert wurden. Wir verwendeten multiple lineare Regressionen, um die Zusammenhänge zwischen Feedbackdaten, Gesprächsthemen und Lernzielen zu ermitteln.

**Ergebnisse:**

Themen wurden in den Feedbackgesprächen eher als Stärken diskutiert, wenn die skalenbasierten Bewertungen hoch waren und es viele positive Kommentare zu einem Item gab. Themen wurden eher als Bereiche zur Verbesserung diskutiert, wenn die Anzahl der negativen Kommentare hoch war. Themen mit vielen (positiven und negativen) Kommentaren und Themen, die als verbesserungswürdig diskutiert wurden, führten eher zu Lernzielen. Wir fanden eine Reihe von Lernzielen, die über die Kompetenzen des MSF-Fragebogens hinausgehen und im Zusammenhang mit der Entwicklung der professionellen Identität gesehen werden können.

**Fazit:**

Da das Feedback und die Lernziele über die im MSF-Fragebogen aufgelisteten Kompetenzen hinausgingen, scheint es uns notwendig, diese umfassenderen Themen, die die Entwicklung der Weiterzubildenden betreffen, anzusprechen. Daher empfehlen wir Supervisionspersonen und Weiterzubildenden, Aspekte der Entwicklung der professionellen Identität wie Karrierepläne, Engagement in der Forschung oder persönliche Haltungen ausdrücklich in regelmässige Bewertungen und Feedbackgespräche einzubeziehen. Auf diese Weise könnte MSF ein geeignetes Instrument sein, um die Entwicklung der professionellen Identität zu unterstützen.

## 1. Introduction

Multisource Feedback (MSF), or 360° assessment, is used to obtain specific feedback to support medical training, mainly the development of skills and competences [1], [2], [3], [4]. In this article we argue how MSF might also be used to support Professional Identity Formation as described by Jarvis-Selinger [5]. Unlike other workplace-based assessments, MSF includes several perspectives on a resident’s performance in various situations over time. In some settings of MSF, a supervisor summarises and “transmits” the feedback, including all scale-based ratings and written narrative comments, to the resident. During this feedback conversation, they compare the feedback to the resident’s self-assessment and define the resident’s learning goals, guiding each resident individually.

Most studies on MSF focused on consultants and specialists as recipients of the feedback and investigated factors that influence the effectiveness and outcomes of MSF [6], [7], [8], [9], [10], [11]. Despite recommendations for feedback conversations to guide emotional responses [11] and reflection [9], few studies have investigated factors influencing MSF in settings where these conversations actually occurred [6], [7], [9], [11]. Some studies based the need for feedback conversations on participant expectations rather than experiences [8], [10], [12]. No study analysed the content or process of these conversations, or observed the facilitators and residents interacting. We thus lack understanding of how facilitators filter and summarize MSF ratings or use them to create learning goals. In non-medical settings, where feedback was given without a conversation, the learning goals were shaped by the number of narrative comments, the polarity of comments (favourable or unfavourable), and the focus of comments (task-focused versus trait-focused) [13].

A better knowledge on the content of the feedback conversation between supervisor and resident is needed to understand how learning goals are set and what they refer to. In this study, we investigate how MSF ratings influenced the feedback conversation and how both shaped the number and content of the resident’s learning goals. We aim to better inform the training of supervisors and residents to improve the targeted use of MSF.

## 2. Methods

### 2.1. Ethics

The local committee of the Association of Swiss Ethics Committees deemed the study exempt from further approval. All participants gave written informed consent to participate and publish their anonymised data.

### 2.2. Setting 

MSF was conducted at the Department of Surgery of the University Children’s Hospital Zurich, Switzerland, where it is a mandatory part of residency training with a formative purpose. Implemented in 2015, based on best-practice literature [6], [7], [9], [14], [15], [16], [17], [18], [19], [20], all participants (residents, raters and supervisors) were taught the objectives, content, and the MSF questionnaire, including the rating scale and feedback rules. Supervisors learned to give specific feedback while preserving rater anonymity. The MSF questionnaire, was based on the Can-MEDS roles [21] and previously described in detail [22].

The MSF process in this study involved up to 15 raters from predefined groups of co-workers providing feedback twice a year. Raters completed the MSF questionnaire online with feedback later presented anonymously as part of a summary with other raters’ feedback. During the feedback conversation the supervisor shared the means of the scale-based ratings with the resident and summarised anonymised narrative comments they felt were important. The supervisor and the resident decided which items on the MSF questionnaire to discuss, set the topics of the feedback conversation, and decided of the number and content of learning goals. They documented MSF ratings, strengths or areas for improvement, and the learning goals on a structured form. 

### 2.3. Study design and data collection

To address the specific literature gap around the feedback conversation, we deliberately decided for a quantitative study design. To trace the way of data during the turn of the conversation as exactly as possible, we disassembled the conversation into three smaller steps, each of which containing documented, quantifiable data. 

This study comprised 75 sets of MSF for residents, each consisting of 12-15 external ratings per residents and one self-rating per resident. Data included 1015 external ratings and 75 self-ratings. As ten sets missed learning goals, only 65 sets of MSF could be analysed. More detailed information and an overview on the three components of the feedback conversation are shown in table 1 (Tab. 1).

### 2.4. Definition and calculation of narrative comments, learning goals, and influencing factors

Data coding was based on [13] and [23].

#### 2.4.1. Polarity of narrative comments

Narrative comments that clearly praised or reinforced were coded “favourable”. Corrections and critical comments, including those made in a critical tone (“sometimes too motivated”, “not sure, if knowledge is enough”) were coded “unfavourable”. Comments that could not clearly be sorted were coded “cannot be allocated”.

#### 2.4.2. Quality of narrative comments

Narrative comments were also coded as task-focused or trait-focused. Comments that clearly described the way a certain task had been or should be conducted, were coded “task-focused”. Comments that directly commented on the person or their attitudes were coded “trait-focused”. Comments that could not clearly be sorted were coded “cannot be allocated”.

#### 2.4.3. Number of learning goals

We defined and counted learning goals based on their content. We had access to 75 sets of minutes from feedback conversations but excluded 13 of them from the analysis of learning goals because they contained no notes on learning goals (these residents were at the end of their training at the department). Notes from two other conversations were lost, so our data set included the text of 60 of the 75 sets of minutes.

#### 2.4.4. Content of learning goals

We checked to see if each documented learning goal could be assigned to one or more items on the MSF questionnaire. Goals associated with more than one item (e.g., “acquisition of knowledge”) or goals that did not seem to be connected to the MSF questionnaire were analysed qualitatively and counted separately. We then assigned all learning goals to CanMEDS roles (see table 2 (Tab. 2)).

#### 2.4.5. Influencing factors

We calculated influencing factors based on 1015 scale-based external ratings and 75 scale-based self-ratings. All 16 items of the MSF questionnaire were rated on a 5-point scale, from “below my expectations” (1) to “far above my expectations” (5), or alternatively “unable to comment” with space provided for narrative comments directly after each item.

### 2.5. Regression analyses

We used multiple linear regression analyses to determine the influence of scale-based external ratings, the number of comments, the number of favourable comments, the number of unfavourable comments and the gap between external ratings and self-ratings. To check that the assumptions for multiple linear regressions were met, we used the Breusch-Pagan test for analysing homoscedasticity, the VIF value for analysing multicollinearity, and the Shapiro-Wilk test for normal distribution of residuals. As all three models showed a significant Breusch-Pagan test indicating heteroscedasticity we used heteroscedasticity consistent standard errors. In all three models VIF values were below 10, indicating no multicollinearity. According to the Shapiro-Wilk test residuals were normally distributed. Results of the statistical analysis can be found in table 2 (Tab. 2).

We displayed the unstandardized beta (Β), the standard error for the unstandardized beta (SE(Β)), the standardized beta (β), the t-test statistic (t), and the probability value (p). Our regression analyses first investigated the influence of those variables on the discussion of strengths, and, second, on the discussion of areas of improvement, and third, on learning goals set during the feedback conversation. As an index of effect size, we report R^2^. The level of significance was set at p<0.05. All analyses were conducted using R (version 3.2.0) [24].

## 3. Results

### 3.1. Narrative comments

MSF data included 3024 narrative comments, averaging 2.63 comments per item per resident (between 0 and 12, SD=1.90) and 39.51 comments in sum per resident (between 15 and 66, SD=12.09). Of the narrative comments, 77% were favourable, 15% were unfavourable, 7% could not be allocated; 95% of narrative comments were task-focused, 0,9% trait-focused, and 3,5% could not be allocated. 

### 3.2. Learning goals

#### 3.2.1. Number of learning goals

In total, we analysed 132 separate learning goals, averaging 2.13 (between 0 and 4) learning goals per resident per feedback conversation.

#### 3.2.2. Content of learning goals

**Figure 1:**
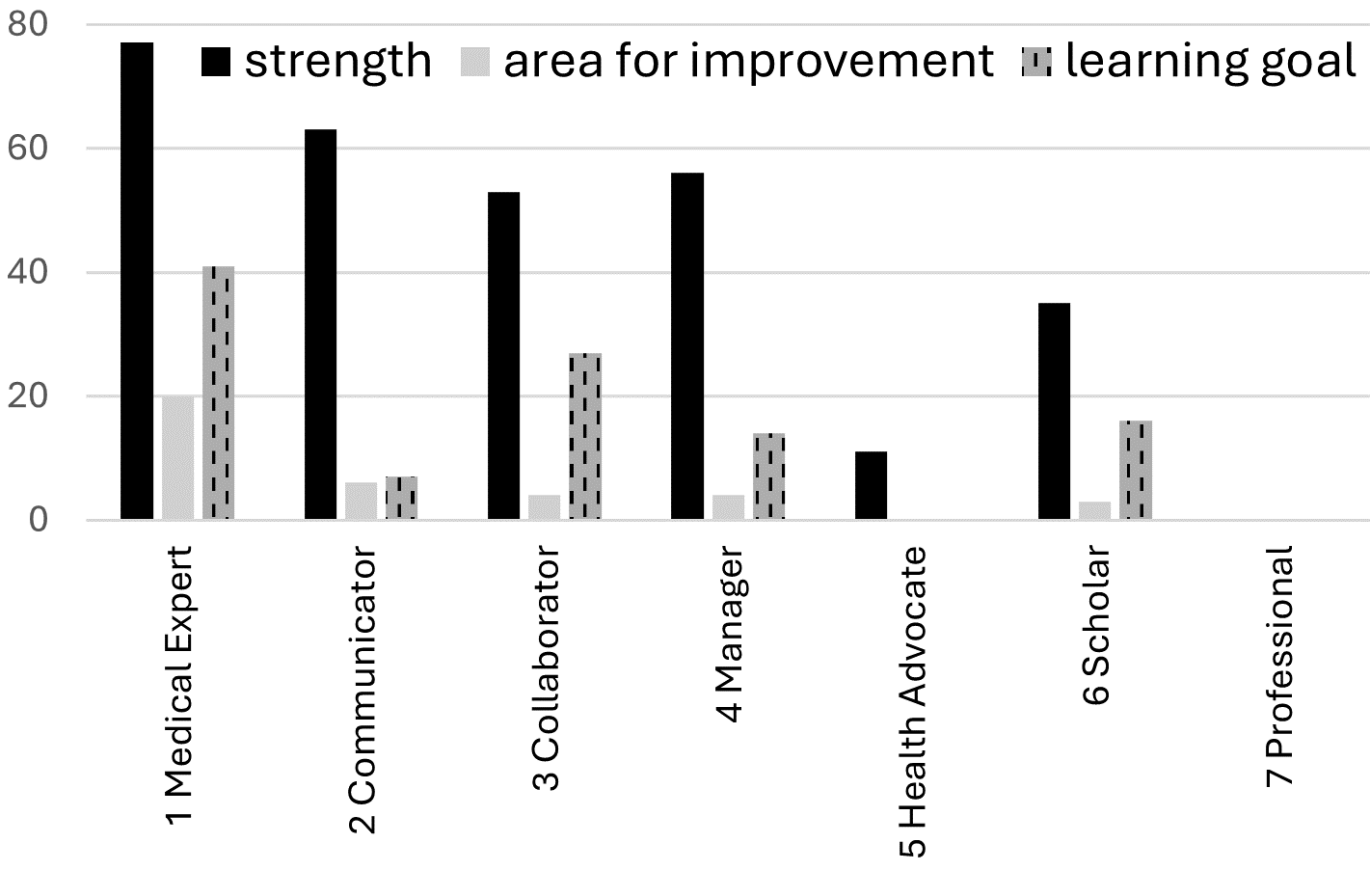
Content of the conversations and learning goals, documented on the structured form for the feedback conversation, dependent on CanMEDS roles Note: this chart excludes the 3 goals that focused on overall performance and the 24 career planning goals.

Of these 132 goals, 68 could be matched directly to single items on the questionnaire, 64 matched multiple items or referred to topics not mentioned in the MSF questionnaire. These 64 goals were of different quality compared to the competence-based items of the questionnaire and addressed broader concepts that can be understood as referring to Professional Identity Formation. They concerned career planning (n=24), gaining knowledge (n=15), scientific work (n=9), appearing self-confident (n=7), working independently (n=3), and other topics (e.g. language skills, showing presence, dealing with stress; n=6). To integrate as many goals as possible into the calculations, we collated them, despite three overall goals and 24 goals on career planning, to the related CanMEDS roles, see table 3 (Tab. 3) and figure 1 (Fig. 1).

### 3.3. Influencing factors

**Figure 2:**
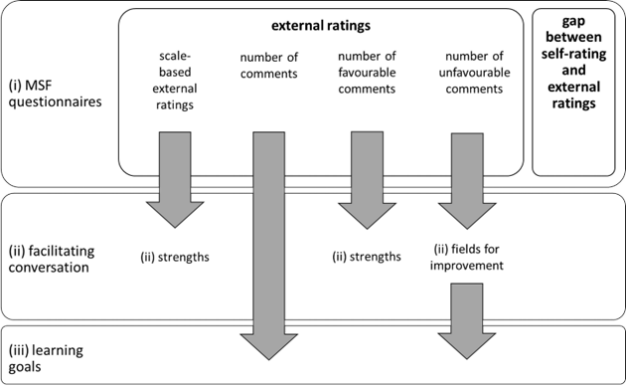
Overview of factors that influence the feedback conversation and the learning goals Arrows represent significant influence.

Our findings are summarised in figure 2 (Fig. 2).

When we analysed influencing factors, we found significant regression equations (F(5/1116)=48.3, p<0.05) with an R^2^ of 0.17 for topics discussed as strengths (F(5/1116)=27.5, p<0.05), an R^2^ of 0.11 for topics discussed as areas for improvement (F(7/352)=7.8, p<0.05), and an R^2^ of 0.11 for learning goals, see table 3 (Tab. 3), table 4 (Tab. 4) and table 5 (Tab. 5) for regression coefficients.

#### 3.3.1. Number of task-focused or trait-focused comments

Of the narrative comments, 95.5% were task-focused; 1% were trait-focused, and 3,5% could not be allocated. Because of this distribution we decided not to add calculations about the differences between the number of task-focused or trait-focused comments to our calculations of the overall number of comments.

### 3.4. Influences of MSF ratings (part i) on strengths discussed during feedback conversations (part ii)

#### 3.4.1. Scale-based external ratings

Higher scale-based external ratings per item (Β=0.39, β=0.30, p<0.05) significantly increased the likelihood the topic would be addressed as a strength.

#### 3.4.2. Favourable comments

A higher number of favourable comments per item (Β=0.05, β=0.18, p<0.05) significantly increased the likelihood the topic would be discussed as a strength.

The numbers of overall comments (Β=0.00, β=0.00, p=1.00), the number of unfavourable comments (Β=0.00, β=0.01, p=0.90), and the gap between external ratings and self-rating (Β=0.03, β=0.04, p=0.11) did not have significant influence on whether a topic was discussed as a strength (see table 4 (Tab. 4)).

### 3.5. Influence of MSF ratings (part i) on topics discussed as areas for improvement during the feedback conversation (part ii)

#### 3.5.1. Unfavourable comments

A higher number of unfavourable comments per item (Β=0.06. β=0.23. p<0.05) significantly increased the likelihood the topic would be discussed as an area for improvement. Scale-based external ratings (Β=-0.03. β=-0.06. p=0.12), overall number of comments (Β=0.02. β=0.15. p=0.07), the number of favourable comments (Β=-0.02. β=-0.13. p=0.10), and the gap between external ratings and self-rating (Β=-0.01. β=-0.04. p=0.14) did not have significant influence on whether a topic was discussed as an area for improvement (see table 5 (Tab. 5)).

### 3.6. Influences of MSF ratings (part i) and topics discussed (part ii) on learning goals (part iii)

#### 3.6.1. Number of comments

A higher number of comments per item significantly increased (Β=0.04. β=0.43. p<0.05) the likelihood the topic would be translated into a learning goal.

#### 3.6.2. Discussed as an area for improvement

Discussing a topic as an area for improvement (Β=0.029. β=0.19. p<0.05) significantly increased the likelihood the topic would be translated into a learning goal.

Scale-based external ratings (Β=-0.06. β=-0.04. p=0.52), the number of favourable comments (Β=-0.03. β=-0.21. p=0.21), the number of unfavourable comments (Β=-0.01. β=-0.03. p=0.76), the gap between external ratings and self-rating (Β=0.01. β=0.01. p=0.88), and discussing a topic as a strength did not significantly influence its translation to a learning goal (Β=0.01. β=0.01. p=0.79) (see table 6 (Tab. 6)).

## 4. Discussion

Our investigation revealed that topics discussed as strengths depended on scale-based ratings and the number of favourable comments while areas for improvement depended on the number of unfavourable comments. Gaps between self-ratings and external ratings did not increase the likelihood of discussion. Each learning goal was influenced by the number of narrative comments and whether the topic had been discussed as an area for improvement. Unexpectedly, we found a range of narrative comments and learning goals that exceeded the competences suggested on the MSF questionnaire and related to broader concepts. We see those goals connected to professional identity formation, discuss them under 4.2. and propose implications for practice based on this finding.

### 4.1. Narrative comments

The high proportion of favourable narrative comments aligns with other studies [23], [25]. We found a higher proportion of task-focused comments [13], [23], likely due to differences in rater training or questionnaire design. Both Dory et al. [26], who studied in-training assessment reports based on written feedback, and Lockyer et al. [23], who studied MSF, indicate that even small adjustments in the format of the questionnaire can increase the quality of feedback. We concluded that the specific rater training and item formulations, asking for task-focused comments, raised the proportion of task-focused comments. 

### 4.2. Learning goals and connection to Professional Identity Formation

We observed between 1 and 4 learning goals per feedback conversation. Many of these learning goals could not be clearly tied to single items of the MSF questionnaire; some addressed combinations of items, and others addressed aspects of the residents’ development not covered by the questionnaire, e.g., career plans, engagement in research or personal attitudes like appearing more self-confident. This finding can be interpreted through the lens of professional identity formation theory [5], which explains that during a resident’s development, the focus of learning goals might shift from single, separate skills to broader, more personal goals. Thus, MSF conversations with residents might be expanded to encompass both *development* goals and *learning* goals.

Our finding that some learning goals seemed unrelated to items on the MSF questionnaire hints at the need to adjust the questionnaire to meet learners’ additional needs. These additional learning goals might also indicate gaps in the curriculum. An analysis of learning goals could be used to actively improve the residency curriculum [27], by including overarching goals.

When learning goals exceed the scope of the MSF questionnaire, it points out the complexity of measuring the effectiveness of MSF since the MSF process addresses both competences and overarching goals. Orienting the feedback conversation to meet these additional goals might not directly increase the effectiveness of MSF as a formative assessment of competences, but expand its range. Embracing the added use might exceed the assessment of competences and might support professional identity development. The MSF could better fulfil this second aim by extending the training of supervisors and residents to encompass the added content and prepare them to better formulate these overarching goals. To see whether this also holds true in an international context, more cross-validation is needed, as MSF is idiosyncratic in every setting, surrounded by different learning cultures including other forms of assessment in different curricula of residency training.

### 4.3. Influencing factors

Items were more likely to be discussed as strengths with more favourable comments and as an area for improvement with more unfavourable comments. Higher ratings predicted discussion of strengths, but lower ratings did not predict discussion of improvements; only the number of unfavourable comments did. This is in contrast to studies by Sargeant et al. [7] and Overeem et al. [15], who reported low ratings as a main motivator for change. A recent qualitative study [28] described that feedback conversations, perceived as summative, affected the residents’ behaviour leading them to conceal weaknesses. A review of the assessor’s behaviour [29] found this group also felt this tension, which led them to avoid delivering unpleasant results. This might partially explain our findings that low ratings and the gap between external ratings and self-ratings were not discussed as often as high ratings. Focussing on the formative aspect of feedback might lower this barrier to honest conversation [29].

Also, our study found significant differences between external and self-ratings, as other studies did, but we did not confirm the influence of this difference on conversation or the learning goals [15], [18]. Again, it is possible that supervisors avoided directly pointing out the differences to reduce friction during the feedback conversation.

A study of managers by Smither and Walker [13] found that a small number of unfavourable task-focused comments had the strongest effect on performance, but a high number of unfavourable comments was discouraging. The number of written comments in their study averaged 6.8, while we observed even 15 to 66 unfavourable comments per resident. We hypothesise that in our study the supervisors prevented residents from being overwhelmed by too many comments. 

Despite the high number of favourable narrative comments, feedback conversations focused more on areas for improvement, possibly because corrective feedback was felt to be more actionable than reinforcing feedback. A model on reflection supports this argument; Sargeant et al. [11] found that feedback inconsistent with self-perception leads to longer lasting reflection; the resident and supervisor might have unconsciously been engaging in this process. Besides, supervisor training in our study focussed on a balance between strengths and areas for improvement, but did not focus on reinforcing goals.

### 4.4. Implications for practice

#### 4.4.1. Professional Identity Formation

We suggest expanding the MSF questionnaire and participant training to meet the residents’ need for overarching development goals. By addressing both specific competences and broader developmental goals, MSF could support professional identity development, besides assessing competences.

#### 4.4.2. Format of the MSF questionnaire and rater training

For the more clearly defined competences, we encourage the use of clear task-related items to achieve high quality feedback with specific task-focused comments.

#### 4.4.3. Feedback conversation

We suggest a formative focus in feedback conversations to encourage honest discussions and reduce tension. When supervisors and residents decide which topics to discuss, they should combine scale-based ratings with narrative comments because both provide valuable information for learning goals. 

### 4.5. Limitations and strengths of the study

We were limited by our inability to observe the conversation directly; we analysed only data going into the feedback conversation, limited notes, and the learning goals as its outcome. Because our study focused in detail on the quantitative data of MSF, we could not study other factors that also influenced the process like the perceptions of the residents or their emotional response to the feedback.

Our unique approach of breaking MSF into components helped us understand the interplay between scale-based ratings, narrative comments, feedback conversations, and learning goals, elucidating the complex process. Since few studies quantitatively investigated influences on the feedback conversation, and the way feedback was translated into learning goals, our data provides valuable insights. As a quantitative study can only show connections between data, but not explain them, qualitative studies are needed to investigate the residents’ and supervisors’ perspective on our findings and recommendations.

## 5. Conclusion

Our study focused on the feedback conversation, a crucial element in the MSF process. We found that a part of the learning goals set during the conversation concerned the professional development of residents. Thus we suggest to use MSF not only as from of a formative assessment of competences but as a guidance process that helps residents develop their professional identity. We encourage supervisors and residents to explicitly include Professional Identity Formation topics such as career plans, engagement in research or personal attitudes into regular assessments and feedback conversations. Our insights offer practical recommendations for improving the use of MSF and shows a way how Professional Identity Formation might be supported during residency training.

## Notes

### Authorship

The authors Kathrin Neuhaus and Sören Huwendiek share the last authorship.

### Authors’ ORCIDs

- Eva K. Hennel: [0000-0002-7625-5785]
- Felicitas-M. Lahner: [0000-0001-6697-3698]
- Noemi Zweifel: [0000-0001-7313-3929]
- Sigrid Harendza: [0000-0002-7920-8431]
- Kathrin Neuhaus: [0000-0003-2438-1779]
- Sören Huwendiek: [0000-0001-6116-9633]

## Competing interests

The authors declare that they have no competing interests. 

## Figures and Tables

**Table 1:**
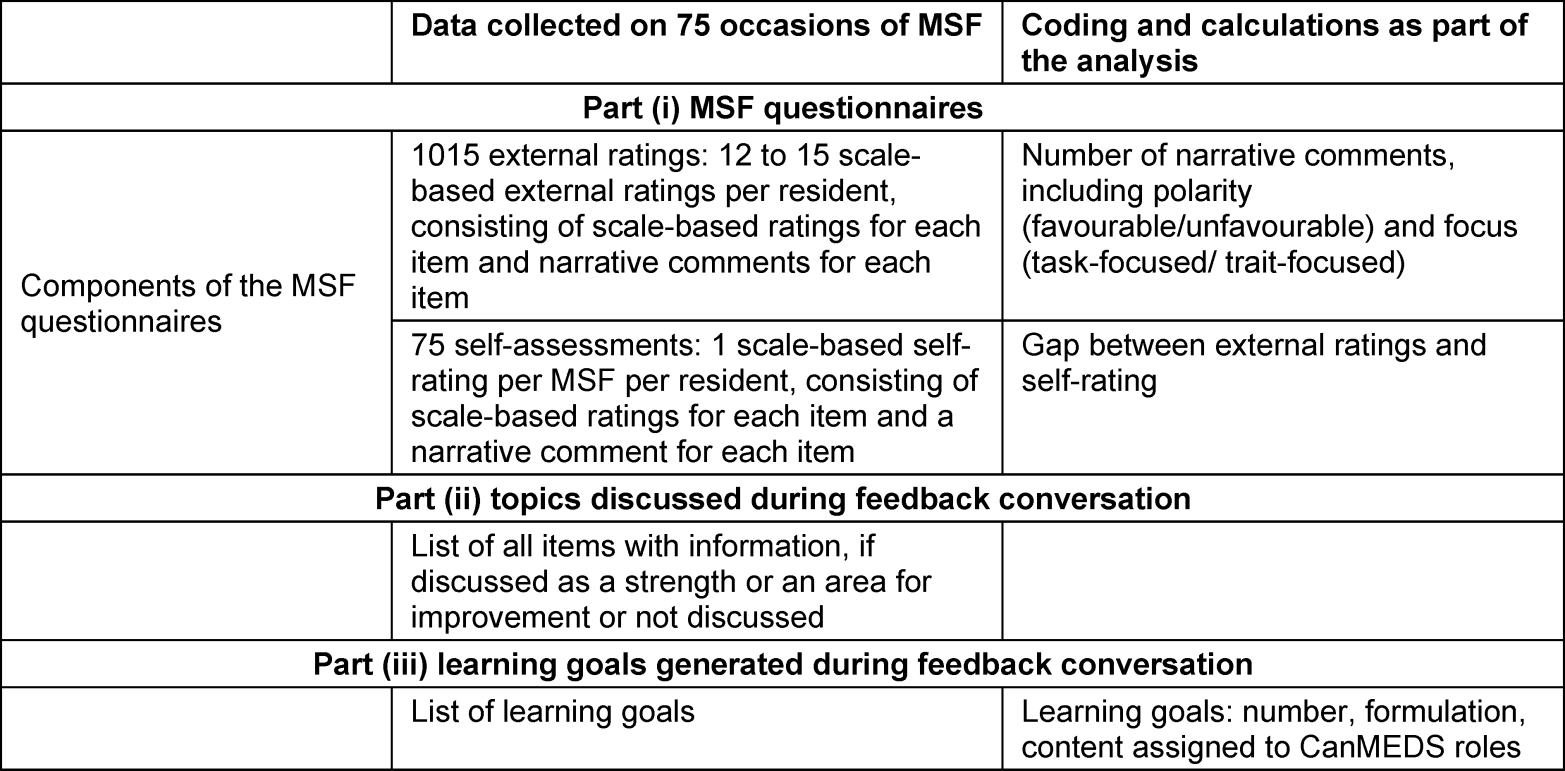
Data collection and analyses according to the three parts of MSF investigated in this study

**Table 2:**
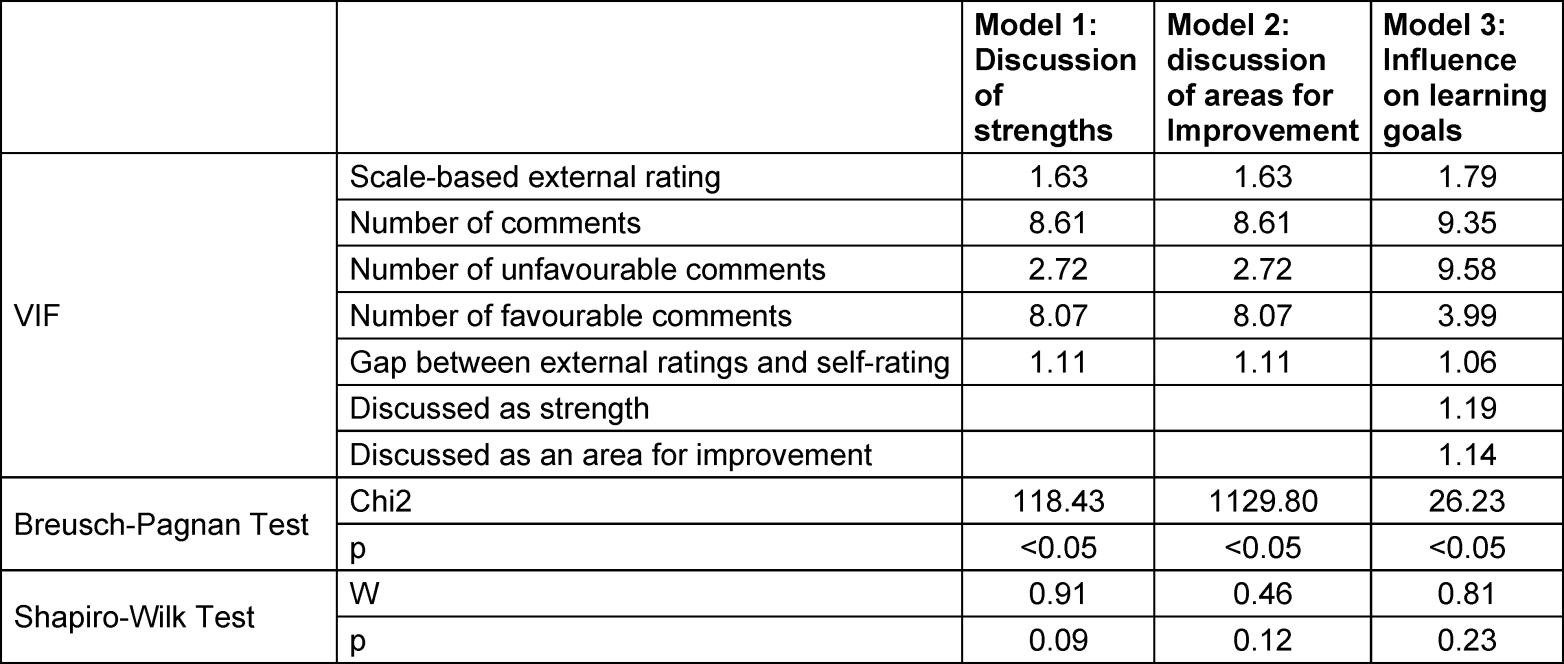
Assumptions for multiple linear models

**Table 3:**
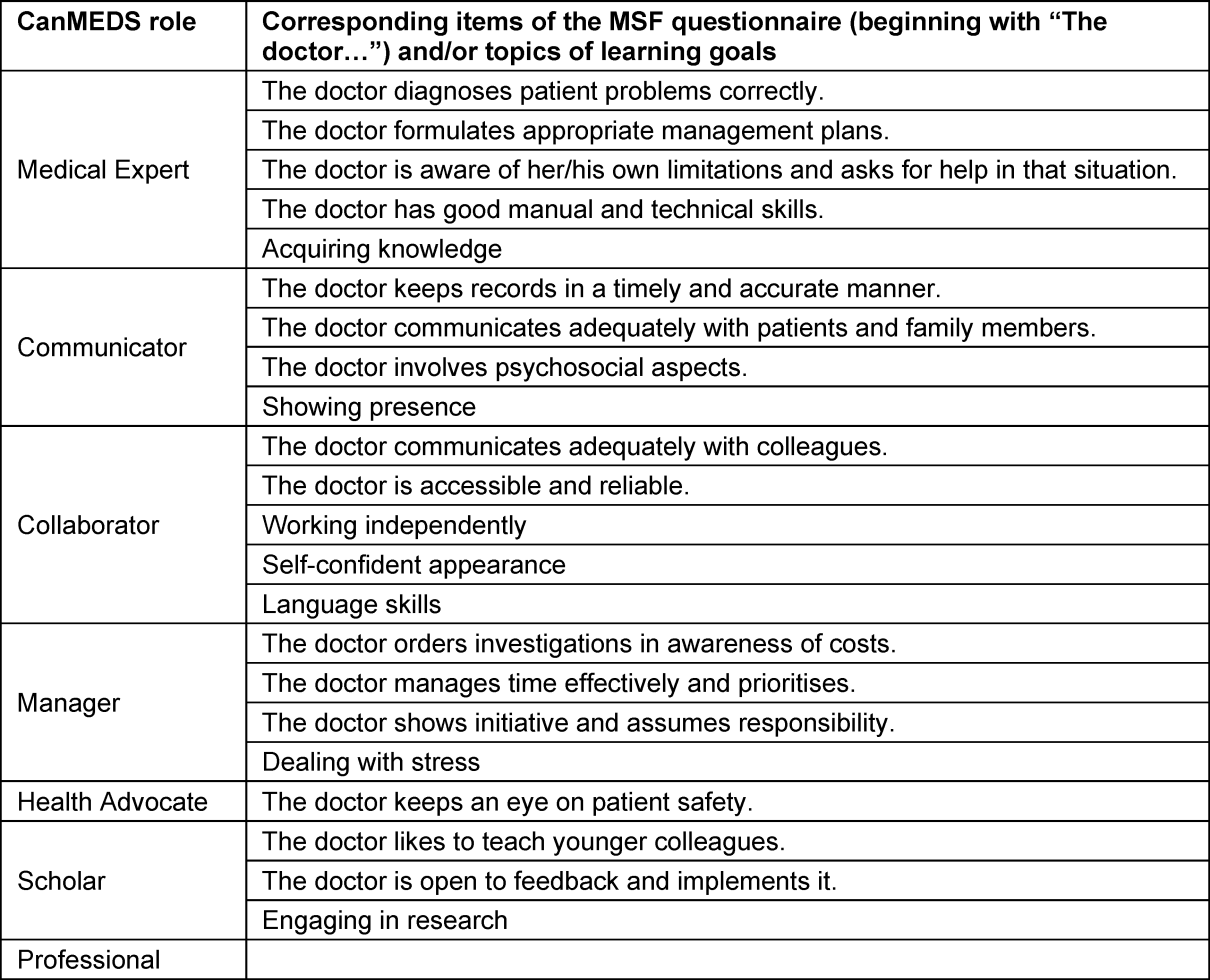
Allocation of items of the MSF questionnaire and learning goals to CanMEDS roles Three goals which concerned the overall performance and 24 goals which concerned career planning were not allocated and not included into the calculations

**Table 4:**
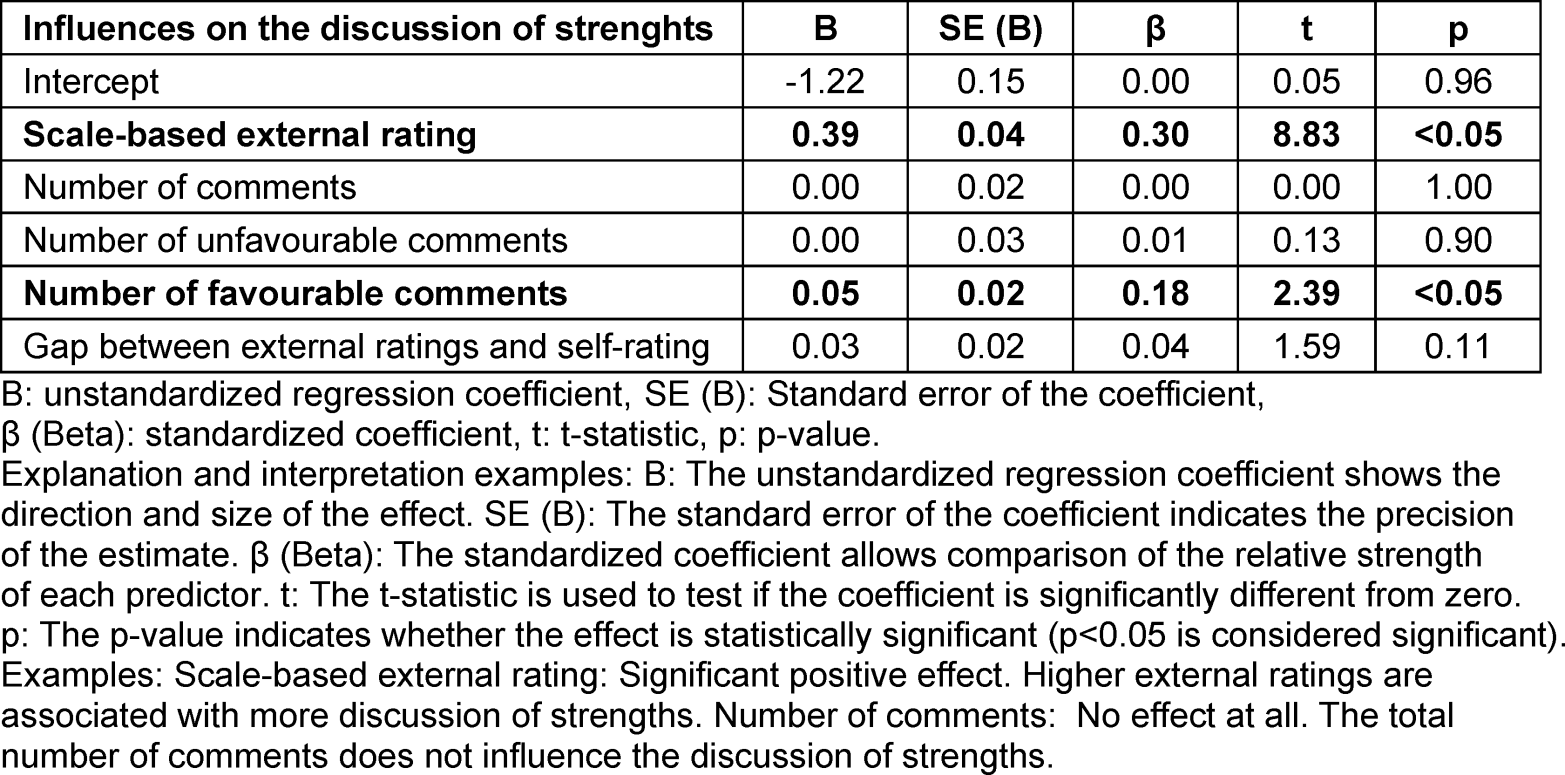
Influences on the discussion of strengths

**Table 5:**
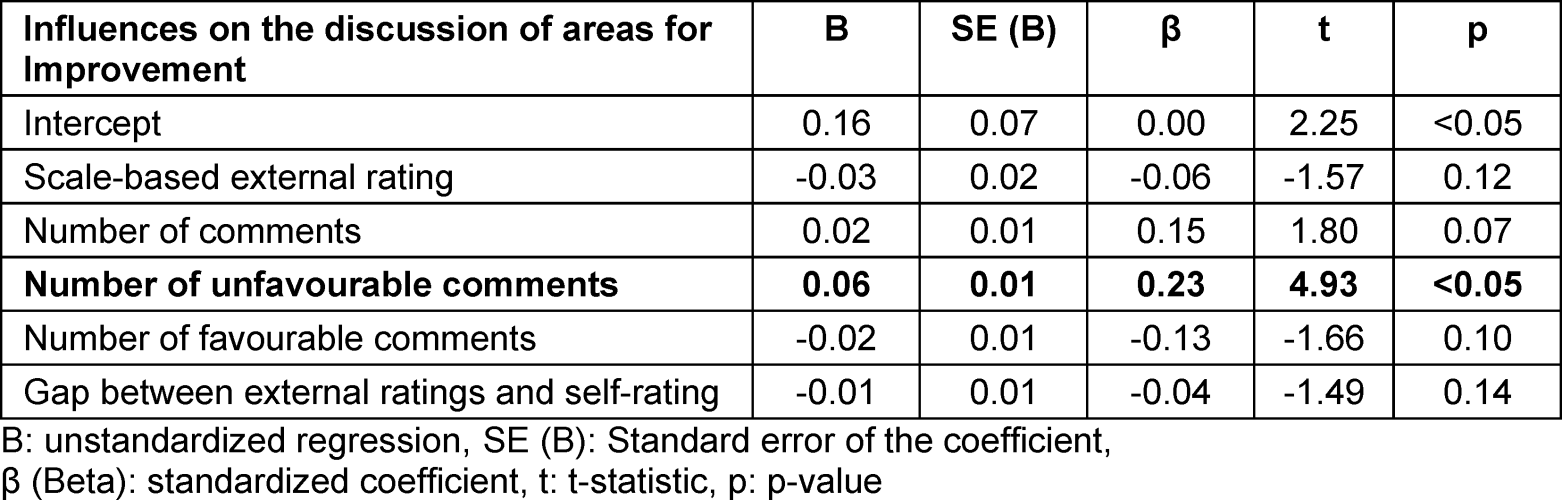
Influences on the discussion of areas for improvement

**Table 6:**
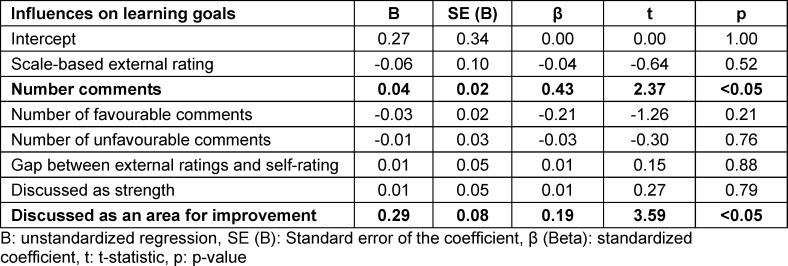
Influences on learning goals
